# Summation and Cancellation Effects on QRS and ST-Segment Changes Induced by Simultaneous Regional Myocardial Ischemia

**DOI:** 10.3389/fphys.2018.00275

**Published:** 2018-04-03

**Authors:** Miquel Vives-Borrás, Esther Jorge, Gerard Amorós-Figueras, Xavier Millán, Dabit Arzamendi, Juan Cinca

**Affiliations:** Department of Cardiology, Hospital de la Santa Creu i Sant Pau, Institut d'Investigació Biomèdica - Sant Pau, CIBERCV, Universitat Autònoma de Barcelona, Barcelona, Spain

**Keywords:** myocardial ischemia, QRS complex, ST-segment, coronary artery occlusion, *in situ* heart, pigs

## Abstract

Simultaneous ischemia in two myocardial regions is a potentially lethal clinical condition often unrecognized whose corresponding electrocardiographic (ECG) patterns have not yet been characterized. Thus, this study aimed to determine the QRS complex and ST-segment changes induced by concurrent ischemia in different myocardial regions elicited by combined double occlusion of the three main coronary arteries. For this purpose, 12 swine were randomized to combination of 5-min single and double coronary artery occlusion: Group 1: left Circumflex (LCX) and right (RCA) coronary arteries (*n* = 4); Group 2: left anterior descending artery (LAD) and LCX (*n* = 4) and; Group 3: LAD and RCA (*n* = 4). QRS duration and ST-segment displacement were measured in 15-lead ECG. As compared with single occlusion, double LCX+RCA blockade induced significant QRS widening of about 40 ms in nearly all ECG leads and magnification of the ST-segment depression in leads V1–V3 (maximal 228% in lead V3, *p* < 0.05). In contrast, LAD+LCX or LAD+RCA did not induce significant QRS widening and markedly attenuated the ST-segment elevation in precordial leads (maximal attenuation of 60% in lead V3 in LAD+LCX and 86% in lead V5 in LAD+RCA, *p* < 0.05). ST-segment elevation in leads V7–V9 was a specific sign of single LCX occlusion. In conclusion, concurrent infero-lateral ischemia was associated with a marked summation effect of the ECG changes previously elicited by each single ischemic region. By contrast, a cancellation effect on ST-segment changes with no QRS widening was observed when the left anterior descending artery was involved.

## Introduction

Simultaneous interruption of blood flow in two coronary arteries is a potentially lethal clinical condition (Mahmoud et al., 2015) that may occur in about 2.5% of patients with ST-segment elevation myocardial infarction (STEMI) (Pollak et al., 2009). However, angiographic and anatomical studies suggest that involvement of two or more coronary segments has a higher prevalence. This assumption is based on the finding of multiple complex coronary plaques in 39.5% of patients with STEMI (Goldstein et al., 2000) and on the observation of ruptured plaques in non-culprit vessels in 10.5 to 37.5% of patients with acute coronary syndromes (Rioufol et al., 2002; Kotani et al., 2003; Tanaka et al., 2005). Moreover, autopsy series show that about 50% of patients who suddenly died in the course of an acute myocardial infarction (AMI) presented intraluminal thrombus in different coronary segments (Davies and Thomas, 1984).

The 12-lead electrocardiogram (ECG) stands as one of the most useful clinical tools to recognize and manage patients with acute myocardial ischemia. However, the diagnostic ECG patterns of simultaneous ischemia in different myocardial regions have not yet been delineated, as the available clinical ECG data are based on a limited number of case reports which are summarized in Table 1. Most of reported cases are males from a wide range of age and the right coronary artery (RCA) was the most commonly affected. The trigger event of the double coronary occlusion may have different etiologies, being the coronary plaque rupture and the coronary vasospasm the most frequently alluded. These clinical reports do not allow to ascertain whether the magnitude of the ECG changes induced by a double coronary occlusion results from a summation or a cancellation of those changes elicited separately by each single vessel occlusion. In a previous experimental study in pigs, we found that simultaneous occlusion of the left anterior descending (LAD) and right coronary arteries attenuated the ST-segment elevation and blunted the reciprocal ST-segment depression in the 12-lead ECG (Cinca et al., 2014), but combination of double occlusions involving the three main coronary systems were not explored. On the other hand, the location of the two concurrent ischemic regions could influence the changes in the QRS complex duration.

**Table 1:** Case reports of patients with a double coronary artery occlusion.

**Case report**	**Sex**	**Age**	**Affected coronaries**	**Alluded Trigger event**
Hamada et al., 1989	Female	59	RCA+LAD	Thrombocytosis
Yoshitomi et al., 1998	Male	34	LAD+LCX	Coronary spasm and thrombus formation
Hosokawa et al., 2001	Male	33	RCA+LAD	Plaque rupture and coronary spasm
Meltser et al., 2004	Male	50	RCA+LAD	Cocaine binge
Sia et al., 2008	Male	46	RCA+LAD	Ruptured plaques
Tu et al., 2009	Male	22	RCA+LAD	Low antithrombin III level
Araszkiewicz et al., 2009	Male	51	RCA+LAD	Ruptured plaques
Lee et al., 2010	Male	75	RCA+LAD	Ruptured plaques
Benjelloun Bennani et al., 2010	Male	53	RCA+LCX	Ruptured plaques
Song et al., 2011	Male	52	LAD+LCX	Thrombus, no coronary stenosis
Yagmur et al., 2012	Female	49	LAD+ LCX	Idiopathic thrombocytopenic purpura
Gan et al., 2012	Male	88	RCA+LAD	Ruptured plaques
Ahmed and Abdul, 2013	Male	47	RCA+LAD	Ruptured plaques
Edem et al., 2015	Male	57	RCA+LAD	Ruptured plaques
Karabay et al., 2015	Male	38	RCA+LAD	Ruptured plaques
Atmaca et al., 2018	Female	33	LAD+LCX	Oral contraceptive

*RCA: Right coronary artery; LAD: Left anterior descending artery; LCX: Left circumflex coronary artery*.

Therefore, the aim of this study was to determine the QRS complex and ST-segment patterns of concurrent ischemia in different myocardial regions elicited by combined double occlusion of the three main coronary arteries. We used closed chest anesthetized pigs and expanded the ECG recording to posterior thoracic leads to explore more accurately the various left ventricular (LV) regions.

## Materials and methods

### Ethical approval

The study protocol was approved by the Animal Care and Use Committee of our institution (No. 8988) and conformed to the regulation for the treatment of animals established by the Guide for the Care and Use of Laboratory Animals, 8th ed. (National Research Council. Washington, DC: the National Academies Press, 2010). Swine were housed in an enriched environment maintained on a 12:12 h light-dark cycle at ~21–23°C with fresh tap water and standard chow available *ad libitum*. At the end of the experiment the animals were killed accomplishing the standards required by the European Union legislation, set out in Annex IV in the European Directive 2010/63/EU.

### Study population

Fourteen female domestic swine (Landrace-Large White cross) weighing about 45 kg were premedicated with azaperone (8 mg/kg intramuscular, Stressnil, Esteve Farma SA, Barcelona, Spain) and anesthetized with propofol (2–4 mg/kg intravenously). After endotracheal intubation, general anesthesia was maintained with a mixture of oxygen and sevofluorane (2.5–3.5%) and pulmonary ventilation was supported mechanically. Fentanyl (0.005 mg/kg intravenous, Fentanest, Kern Pharma SL, Barcelona, Spain) was administered during the procedure for analgesia.

### Experimental preparation

#### Coronary artery occlusion

Acute transmural myocardial ischemia was induced by percutaneous coronary catheter balloon occlusion. Both femoral arteries were catheterized, and two 7F introducers (Cordis; Miami;FL) were used to insert 6F guiding catheters (Cordis). The catheters were advanced to the ostium of the left and/or right coronary arteries under fluoroscopic guidance, and a 3 mm diameter catheter balloon (Cordis) was placed and inflated to its nominal diameter at the mid segment of the corresponding coronary arteries. We also verified by coronary angiography, the position of the catheter, the complete occlusion of the mid segment and the presence of appropriate flow to the proximal segment of the artery. Sodium heparin was administered intravenously as a 150 IU/Kg bolus at the beginning of catheter manipulation followed by 100 IU/Kg every 1 h until the end of the study.

#### ECG monitoring

A 15 lead-ECG was continuously recorded and stored on a CardioSoft ECG system (Version 6.7.3, GE Healthcare, Freiburg, Germany). Due to species anatomy, the conventional precordial leads V1–V6 were positioned one intercostal space above that used in current clinical electrocardiography (Cinca et al., 2014). The three additional chest leads V7–V9 were placed equidistantly following the level of lead V6. In each ECG recording we measured the QRS duration and the ST-segment deviation at the J point taking the PR segment as reference. The measurements were taken at baseline and 5 min after occlusion. The parameters were measured with electronic calipers (Cardio-Calipers software, Iconico^©^) under appropriate image magnification.

### Experimental series

The pigs were submitted to single and double coronary occlusions according to the following randomization: Group 1: Left circumflex (LCX) and RCA arteries. Group 2: LAD and LCX, and Group 3: LAD and RCA.

### Study protocol

All animals underwent 5 min occlusion−10 min reperfusion for each of the two selected arteries. Thereafter, both arteries were occluded simultaneously for 5 min. To minimize the preconditioning effect elicited by repeated occlusions the sequence of the single coronary artery occlusion was changed in each animal. Pigs developing ventricular fibrillation were excluded in order to avoid ST-segment distortion related to electrical defibrillation. All animals were free of significant atherosclerotic coronary artery disease as denoted by the coronary angiography performed at the beginning of the study.

### Statistics

The magnitude of the QRS complex duration and ST-segment displacement was expressed as the mean and standard error of the mean (SEM). The changes in the QRS complex duration were evaluated by comparing the baseline values with those reached after single or double coronary occlusion. The ST-segment changes induced by double occlusion were compared with those observed after each single coronary occlusion. The statistical method used to test these differences was the general linear model for repeated measures. The Bonferroni method was used for adjusting the multiple comparisons. A *p*-value < 0.05 was considered significant. All analyses were performed using SPSS v.22.0 (IBM SPSS Inc., Chicago, IL, USA).

## Results

Two pigs developed ventricular fibrillation and were excluded from the study. The 12 remaining pigs entered in the study and were distributed equally among the three groups. The baseline heart rate was similar among all the experimental animals (77 ± 4 bpm). All pigs had a similar coronary dominance and angiographic distribution pattern.

### Changes in QRS complex duration

Detailed data on the changes in QRS complex duration recorded in the 15-lead ECG in all experimental groups are listed in the Supplementary Material.

**Figure 1:**
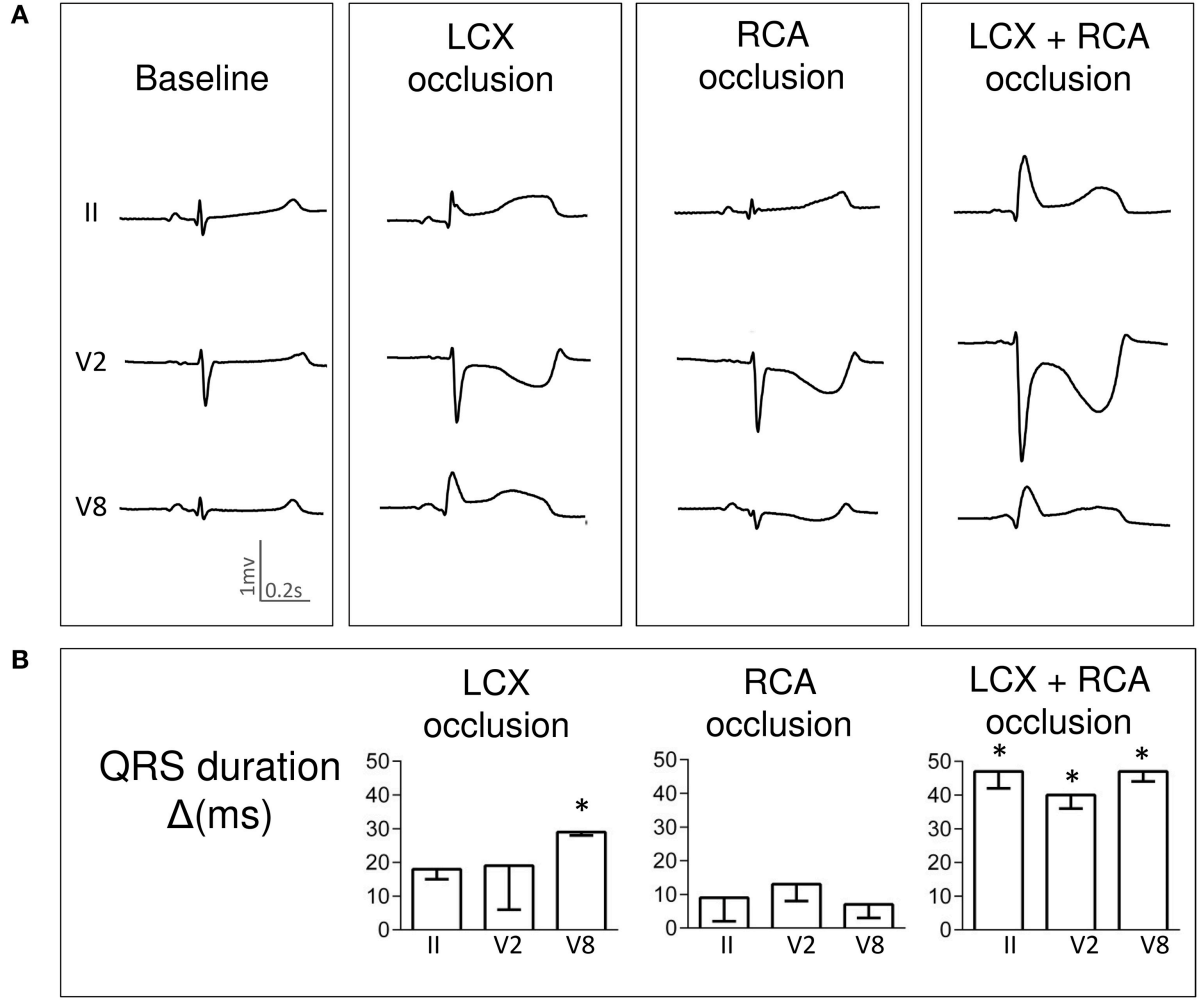
Changes in QRS complex duration induced by single and simultaneous occlusion of the left circumflex and right coronary arteries in anesthetized pigs. **(A)** illustrates the QRS complex widening in ECG leads II, V2 and V8 in a representative case at baseline and after 5-min of occlusion of the left circumflex, right, and double coronary arteries. **(B)** shows the mean (bars) and standard error (whiskers) of the QRS duration changes from baseline (Δ) in ECG leads II, V2 and V8 recorded in the 4 pigs of the left circumflex and right coronary artery group. Asterisks indicate a *p* < 0.05 for the QRS duration changes from baseline. LCX, left circumflex coronary artery; RCA, right coronary artery; ms, milliseconds.

Single LCX occlusion induced a QRS widening of about 20 ms affecting all ECG leads (maximal in leads V6–V9) but this lengthening was negligible after RCA occlusion. Interestingly, a greater QRS lengthening of about 40 ms was observed after simultaneous LCX+RCA occlusion. Figure 1A illustrates a representative case and Figure 1B depicts the QRS changes in three selected ECG leads.

**Figure 2:**
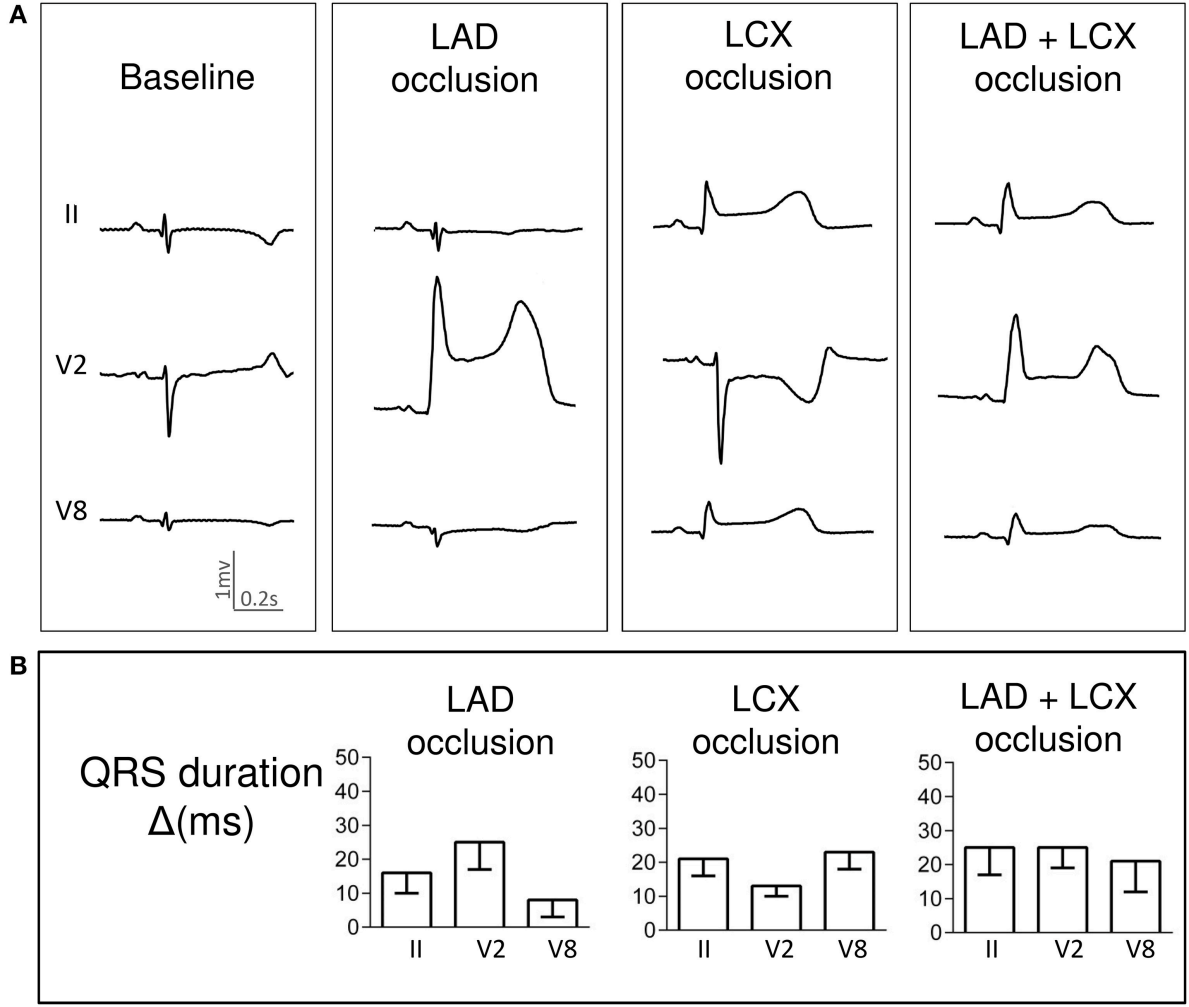
Changes in QRS complex duration induced by single and simultaneous occlusion of the left anterior descending and left circumflex coronary arteries in anesthetized pigs. **(A)** illustrates the QRS complex widening in ECG leads II, V2 and V8 in a representative case at baseline and after 5-min of occlusion of the left anterior descending, left circumflex, and double coronary arteries. **(B)** shows the mean (bars) and standard error (whiskers) of the QRS duration changes from baseline (Δ) in ECG leads II, V2 and V8 recorded in the 4 pigs of the left anterior and left circumflex coronary arteries group. LAD, left anterior descending artery; LCX, left circumflex coronary artery; ms, milliseconds.

**Figure 3:**
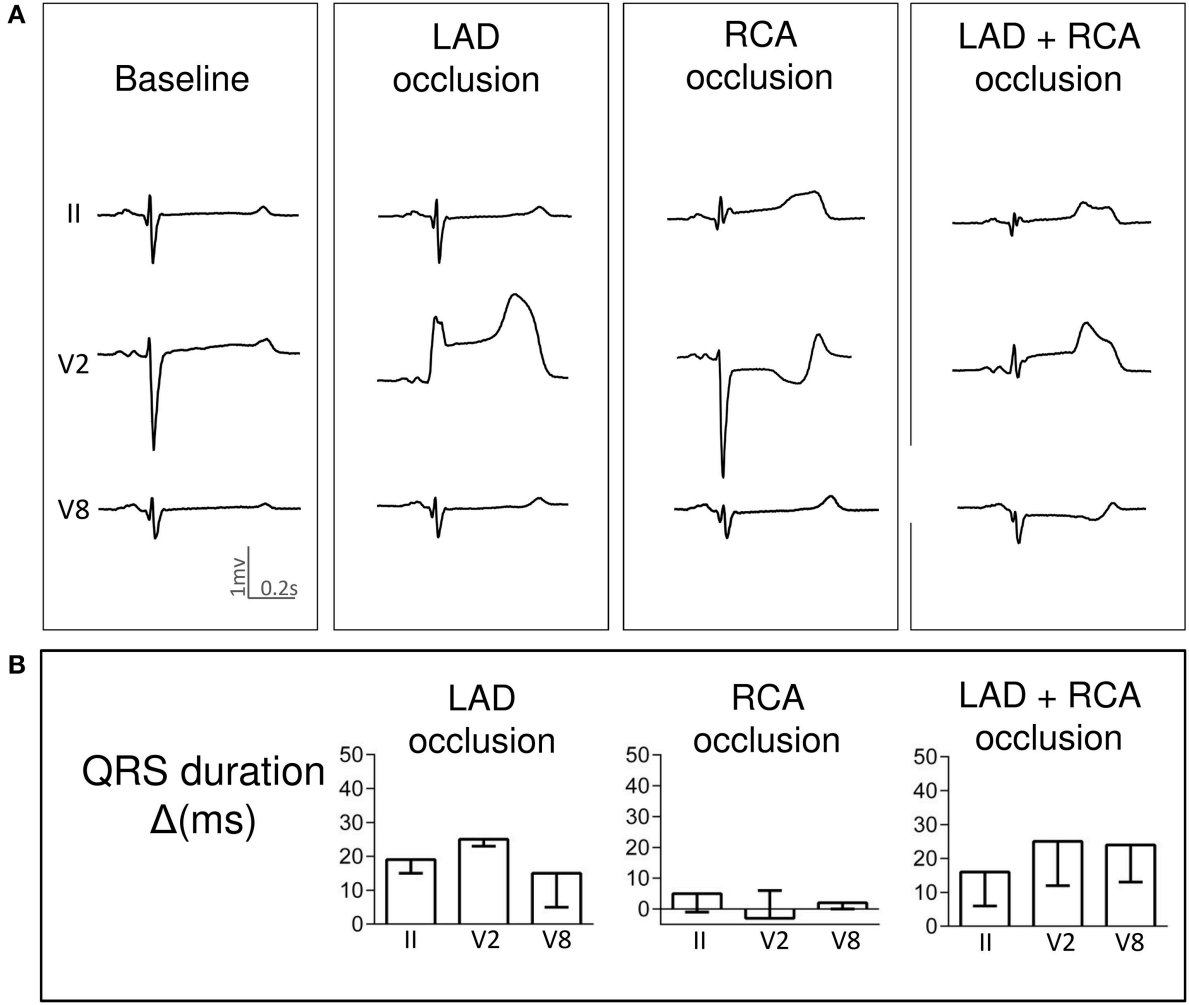
Changes in QRS complex duration induced by single and simultaneous occlusion of the left anterior descending and right coronary arteries in anesthetized pigs. **(A)** illustrates the QRS complex widening in ECG leads II, V2 and V8 in a representative case at baseline and after 5-min of occlusion of the left anterior descending, right, and double coronary arteries. **(B)** shows the mean (bars) and standard error (whiskers) of the QRS duration changes from baseline (Δ) in ECG leads II, V2 and V8 recorded in the 4 pigs of the left anterior descending and right coronary arteries group. LAD, left anterior descending artery; RCA, right coronary artery; ms, milliseconds.

Pigs with LAD occlusion showed a QRS lengthening of about 10–20 ms particularly in precordial ECG leads. Neither simultaneous LAD+LCX nor LAD+RCA ischemia increased significantly the QRS widening attained during the single occlusions. Representative cases are illustrated respectively in Figures 2A, 3A. Likewise, the QRS duration changes in three selected leads are graphically presented in Figures 2B, 3B.

### ST-segment changes

Detailed data on the ST-segment changes recorded in the 15-lead ECG in all experimental groups are listed in the Supplementary Material.

#### LCX and RCA group

**Figure 4:**
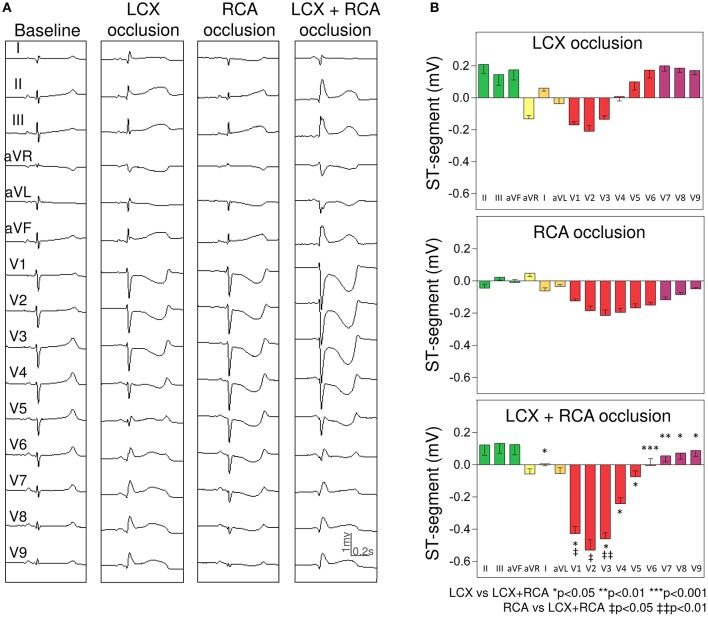
Changes in the ST-segment displacement in the surface 15-lead electrocardiogram induced by single and simultaneous occlusion of the left circumflex and right coronary arteries in anesthetized pigs. **(A)** shows the 15-lead ECG recorded in a representative case at baseline and after single and double 5-min occlusion of the left circumflex and right coronary arteries. **(B)** illustrates the mean (bars) and the standard error of the mean (whiskers) of the ST-segment displacement recorded in the 15-lead ECG in the 4 pigs of left circumflex and right coronary arteries group. From top to bottom the panels illustrate the ST-segment changes induced after left circumflex, right, and double coronary arteries occlusion. LCX, left circumflex coronary artery; RCA, right coronary artery.

As shown in Figure 4, single LCX occlusion induced ST-segment elevation in leads I, II, III, aVF, and V5–V9 (maximal value in lead II: 0.21 ± 0.06 mV) and reciprocal ST-segment depression in leads aVR, aVL, and V1–V3 (maximal depression in lead V2: −0.21 ± 0.03 mV). Occlusion of the RCA induced a diffuse ST-segment depression in leads V1–V9 (more evident in lead V3: −0.22 ± 0.04 mV). Of interest, simultaneous LCX+RCA occlusion was followed by: (i) significant magnification of the reciprocal ST-segment depression in leads V1–V3 (maximal change of 228% in lead V3) and (ii) attenuation of the ST-segment displacement in leads V5–V9 (maximal decrease of 120% in lead V5).

#### LAD and LCX group

**Figure 5:**
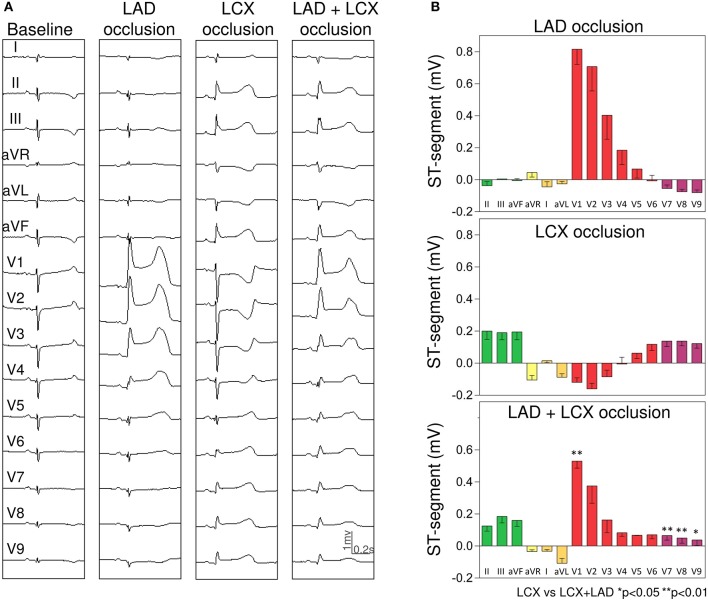
Changes in the ST-segment displacement in the surface 15-lead electrocardiogram induced by single and simultaneous occlusion of the left anterior descending and circumflex coronary arteries in anesthetized pigs. **(A)** shows the 15-lead ECG recorded in a representative case at baseline and after single and double 5-min occlusion of the left anterior descending and left circumflex coronary arteries. **(B)** illustrates the mean (bars) and the standard error of the mean (whiskers) of the ST-segment displacement recorded in the 15-lead ECG in the 4 pigs of left anterior and left circumflex coronary arteries group. From top to bottom the panels illustrate the ST-segment changes induced after left anterior, left circumflex, and double coronary occlusion. LCX, left circumflex coronary artery; LAD, left anterior descending coronary artery.

As illustrated in Figure 5, single LAD occlusion induced ST segment elevation in leads V1–V5 (maximal in lead V1: 0.82 ± 0.09 mV) and reciprocal ST-segment depression in leads V7–V9 (maximal in lead V9: −0.08 ± 0.02 mV). Simultaneous LAD+LCX occlusion damped the ST-segment elevation induced by single LAD occlusion in leads V1–V4 (maximal decrease of 60% in lead V3) and diminished the ST-segment elevation induced by the corresponding LCX occlusion in leads V7–V9 (maximal attenuation of 64% in lead V8).

#### LAD and RCA group

**Figure 6:**
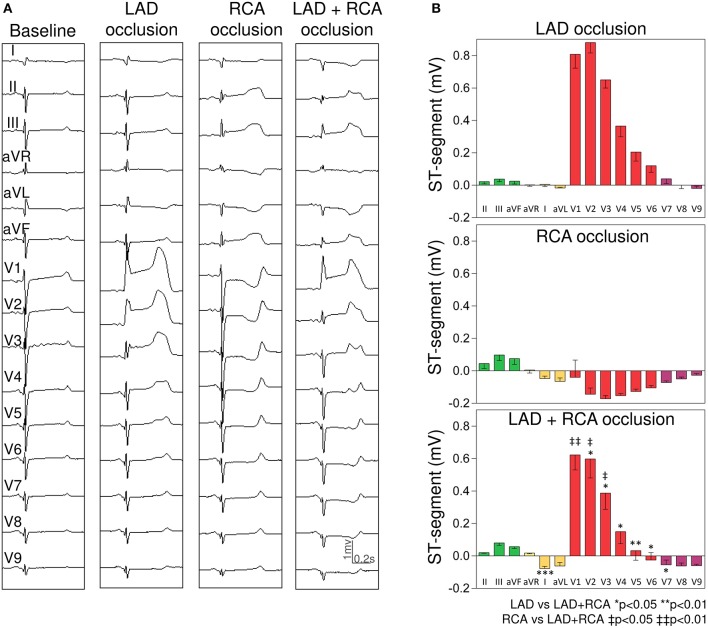
Changes in the ST-segment displacement in the surface 15-lead electrocardiogram induced by single and simultaneous occlusion of the left anterior descending and right coronary arteries in anesthetized pigs. **(A)** shows the 15-lead ECG recorded in a representative case at baseline and after single and double 5-min occlusion of the left anterior descending and right coronary arteries. **(B)** illustrates the mean (bars) and the standard error of the mean (whiskers) of the ST-segment displacement recorded in the 15-lead ECG in the 4 pigs of left anterior descending and right coronary arteries group. From top to bottom the panels illustrate the ST-segment changes induced after left anterior descending, right, and double coronary occlusion. LAD, left anterior descending coronary artery; RCA, right coronary artery.

Concomitant LAD+RCA occlusion (Figure 6) attenuated the ST-segment changes in precordial leads (maximal attenuation of 86% in lead V5).

## Discussion

### Main findings

This is the first systematic description of the acute ECG changes elicited by combined double coronary occlusions involving the three main coronary arteries. Our data revealed that simultaneous occlusion of the LCX and RCA enlarged both the QRS widening and the reciprocal ST-segment changes previously elicited by the single vessel occlusion (summation effect). In contrast, double occlusion of the LAD and either the LCX or the RCA did not enlarge the QRS duration and attenuated the ST-segment changes (cancellation effect). We also found that ST-segment elevation in posterior thoracic ECG leads V7–V9 was a specific sign of LCX occlusion. Thus, our data reveal that the ECG changes induced by double coronary occlusion will be more apparent when simultaneous ischemia involves the infero-lateral rather than the anterior LV regions.

### Changes in QRS complex duration

A major electrophysiological consequence of acute myocardial ischemia is the slowing of the local activation wave front and this is reflected in the surface ECG by a lengthening of the QRS complex duration (Holland and Brooks, 1976). The QRS duration is proportional to the duration of the activation process of both ventricles and therefore ischemia affecting the normally late activated cardiac regions might enlarge the QRS widening. Similar to humans, the latest activated cardiac regions in swine are the posterior and basal segments of the LV wall (Durrer et al., 1970; Gepstein et al., 1997) and indeed we found that pigs with ischemia in these regions (LCX+RCA group) presented the most marked QRS widening. In contrast, when the anterior LV wall was affected (groups LAD+RCA and LAD+LCX), then the QRS widening was not significantly magnified. The largest QRS widening observed during simultaneous infero-lateral ischemia suggests that this clinical condition carries a greater intraventricular dyssynchrony.

### ST-segment changes

**Figure 7:**
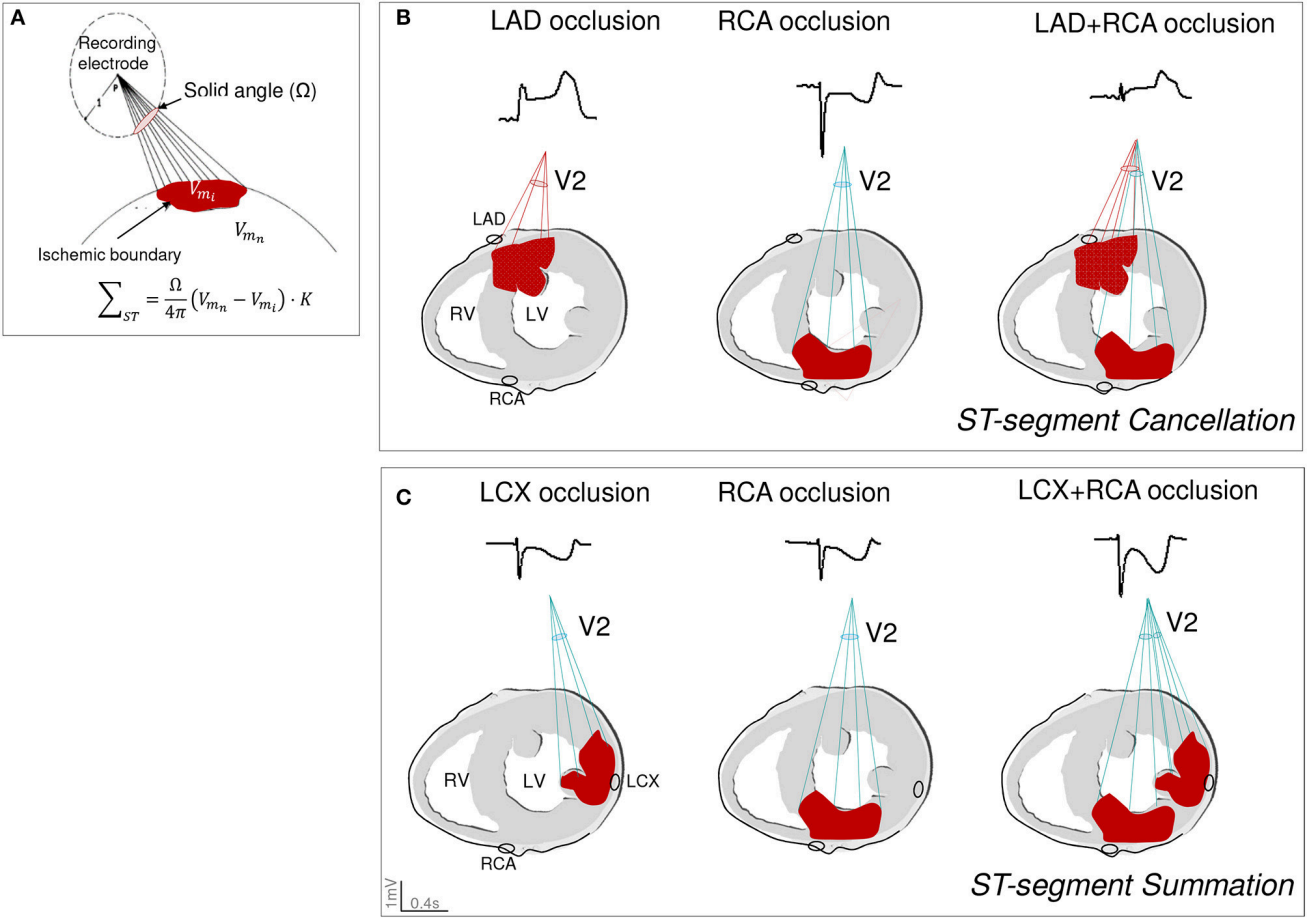
Graphical illustration of the summation and cancellation effects induced by double coronary artery occlusion on the ST-segment changes based on the solid angle theory model. **(A)** reproduces the mathematical and pictorial characterization of the solid angle theory where the magnitude and direction of the ST-segment shift recorded in an electrode location is determined by: the solid angle (Ω) formed by the projection of the boundaries of the ischemic area to the recording site and by the tissue conductivity (K) and the transmembrane potential gradients generated between the normal (Vm_n_) and the ischemic (Vm_i_) areas. Adapted from Holland and Brooks (1977). **(B)** illustrates the ST-segment cancellation effect in lead V2 in a pig with double occlusion of the left anterior descending and right coronary arteries. **(C)** illustrates the ST-segment summation phenomenon in lead V2 in a pig with double occlusion of the left circumflex and right coronary arteries. K, tissue conductivity; Vm_n_, transmembrane potential in the normal tissue; Vm_i_, transmembrane potential in the ischemic areas; LAD, left anterior descending artery; LCX, left circumflex coronary artery; RCA, right coronary artery.

In current clinical practice, the ST-segment patterns are reliable tools to predict the location of the ischemic region in patients with acute coronary occlusion (Noriega et al., 2014). However, the ECG changes induced by simultaneous occlusion of two coronary arteries have not been systematically analyzed. In a previous study in pigs (Cinca et al., 2014) we found that LAD+RCA occlusion attenuated the precordial ST-segment changes induced by each single vessel occlusion (cancellation effect). The present investigation demonstrates that both ST-segment cancellation and summation can occur when the double occlusion protocol is expanded to the three main coronary vessels. ST-segment cancellation was evident in the anterior precordial leads in pigs with LAD+RCA or LAD+LCX occlusion and also in the posterior leads in the LCX+RCA group. In contrast, summation was exclusively observed in pigs with occluded LCX+RCA as in this condition the ST-segment in leads V1–V3 moved in the same direction (downwards) during the single vessel occlusion. The solid angle model (Wilson et al., 1933; Holland and Brooks, 1977) is one among the ECG principles that could explain the summation and cancellation effects during double coronary occlusion. As graphically illustrated in Figure 7A, the magnitude and direction of the ST-segment shift recorded in an electrode location is mainly determined by: (i) the solid angle (Ω) formed by the projection of the ischemic boundary to the recording site (spatial factor), and (ii) the tissue conductivity (K) and the transmembrane potential gradients generated between the normal (Vm_n_) and the ischemic (Vm_i_) areas (non-spatial factors). It is predictable that in the case of simultaneous coronary occlusion, the ST-segment behavior will be more dependent on the spatial relationship of the two ischemic areas with respect to the recording site, rather than on the non-spatial factors. The latter may not influence because ischemia began simultaneously in the two myocardial regions and both would attain similar cell membrane depolarization gradients. Therefore, when the epicardial and endocardial surfaces of the two ischemic areas are counterpoised with respect to the recording electrode the resultant effect is a cancellation of the ST-segment changes. Conversely, a ST-segment summation would be expected when the epicardial and endocardial layers of the two ischemic areas are aligned with the recording electrode. Figure 7B illustrates the ST-segment cancellation effect in lead V2 in a pig with LAD+RCA occlusion and Figure 7C shows the summation phenomenon in the same lead in a pig of the LCX+RCA group.

### Clinical implications

The ECG patterns of double coronary occlusion observed in this experimental model are likely transferable to clinical practice because the electrophysiological derangements induced in the ischemic myocardium in man and pigs are comparable (Cinca et al., 1980) and, moreover, the culprit vessel-related ECG patterns are also similar (Noriega et al., 2013).

Accordingly, our data suggest that the diagnosis of double coronary occlusion can be more reliably established in the presence of simultaneous ischemia in inferior and lateral LV regions because in these circumstances a summation effect is exerted on the QRS duration and ST segment deviation. Consistent with this observation, Pollack et al. reported in their case series of 18 patients that reciprocal changes were more frequently seen in simultaneous LCX+RCA occlusion than in LAD+LCX or LAD+RCA occlusion (Pollak et al., 2009). These findings could be of relevance in patients with concurrent ischemia in the anterior LV region because despite they may present extensive myocardial ischemia, the ECG changes can be attenuated. Also of clinical interest is the observation of increased QRS widening in infero-lateral ischemia since this may contribute to identify patients with greater intraventricular dyssynchrony and hence with less efficient cardiac mechanical work. However, further specific echocardiographic studies should be needed to verify this hypothesis. Combined assessment of the evolving changes in both the QRS duration and the ST segment pattern would be recommended in patients with acute coronary syndromes since this combined approach permitted a better estimation of the myocardial area at risk in patients with STEMI (Vervaat et al., 2014).

ST-segment elevation in the posterior thoracic leads V7–V9 was only observed in pigs with LCX occlusion thus the specificity of these leads to detect lateral LV ischemia further supports the recommendation of recording leads V7–V9 in patients with acute coronary syndromes.

### Study limitations and strengths

The coronary arteries were occluded at their mid third in order to diminish the likelihood of ventricular fibrillation and then avoid excessive exclusion of pigs due to this arrhythmia. A more proximal coronary occlusion could have influenced the ST-segment summation and cancellation patterns described in this study. However, in patients with STEMI we found that proximal and mid occlusion of the RCA or LCX induced similar trend of ST-segment changes and the only differential feature was the occurrence of reciprocal ST-segment depression in inferior leads in patients with proximal LAD occlusion (Noriega et al., 2014). Thus, attenuation (cancellation effect) of the ST segment elevation in inferior leads would be expected when the double coronary occlusion involves the proximal LAD.

In our study, the changes in ST-segment displacement were measured at the first 5 min of ischemia, time at which these changes are fully apparent. Since in clinical practice the STEMI patients are admitted with more evolved myocardial ischemia, the magnitude of the ST-segment changes would be expected to decrease over time and therefore, the ST-segment cancellation effect in patients with anterior ischemia might be more apparent.

The coronary arteries were occluded twice (single and double occlusion) and this may have caused some degree of ischemic preconditioning (Cinca et al., 1992). Although preconditioning may attenuate the ischemic ST segment changes, its influence on the trend of the summation or cancellation patterns reported in this study is unlikely. A major argument is that we previously observed that during double coronary occlusion the selective release of one of the two occluded arteries immediately reverted the ST segment cancellation (Cinca et al., 2014). Moreover, a similar cancellation pattern was observed when the double LAD+RCA occlusion was directly performed without any previous single occlusion in two test pigs. A certain degree of cardioprotection induced by the use of propofol, sevofurane, and opiods cannot be ruled out in our model. However, any background effect of anesthetics on the magnitude of the ischemic ST-segment changes might presumably be present along the entire course of the experiment and would therefore affect all coronary occlusions in the same direction, although not necessarily with the same magnitude. In our study, all coronary occlusions induced noticeable ST-segment changes, thus any potential protective effect of anesthetics was not strong enough to counteract completely the effects of ischemia on the ECG.

During RCA occlusion, the magnitude of reciprocal ST depression in the precordial leads was greater than that of ST elevation in the inferior leads. This finding could likely be explained by the fact that the pig heart apex is directly inferior compared to leftwards in humans thus allowing a preferential projection of the cardiac electrical potentials generated by the RCA occlusion on the precordial rather than the inferior ECG leads.

## Conclusions

Summation and cancellation effects on the ECG patterns are observed during simultaneous ischemia in two LV regions. Concurrent infero-lateral ischemia induced a diffuse QRS widening and ST-segment summation in leads V1–V3. Conversely, when one of the two ischemic areas is the anterior wall, no additional QRS widening is observed and ST-segment elevation is markedly damped. Additionally, leads V7–V9 are reliable tools to detect LCX-related ischemia.

## Author contributions

MV-B and EJ collected, analyzed, and interpreted the data, designed the experiments, and drafted the manuscript. JC along with the co-authors conceived the experiments and revised the manuscript critically for relevant intellectual content. All the authors participated in the experiments. All authors approved the final version of the manuscript and qualify for authorship.

### Conflict of interest statement

The authors declare that the research was conducted in the absence of any commercial or financial relationships that could be construed as a potential conflict of interest.

## Abbreviations

STEMI: ST-segment elevation myocardial infarction
AMI: Acute myocardial infarction
ECG: Electrocardiogram
LAD: Left anterior descending coronary artery
RCA: Right coronary artery
LV: Left ventricular
LCX: Left circumflex coronary artery.
