# Supplementary material for: Summation and Cancellation Effects on QRS and ST-Segment Changes Induced by Simultaneous Regional Myocardial Ischemia
Source: Front Physiol. 2018 Apr 3;9:275. doi: 10.3389/fphys.2018.00275 (PMC5891593; doi:10.3389/fphys.2018.00275)
Supplement: Supplementary file 1 [file Table1.DOCX]

**Supplementary table 1. QRS duration mean difference from baseline (ms) in each ECG lead in the 12 pigs submitted to single and double coronary artery occlusion**

|  | GROUP LCX & RCA (n=4) | | | | | | GROUP LAD & LCX (n=4) | | | | | | GROUP LAD & RCA (n=4) | | | | | |
| --- | --- | --- | --- | --- | --- | --- | --- | --- | --- | --- | --- | --- | --- | --- | --- | --- | --- | --- |
| Leads | LCX | p | RCA | p | LCX+RCA | p | LAD | p | LCX | p | LAD+LCX | p | LAD | p | RCA | p | LAD+RCA | p |
| I | 18 (6) | ns | 2 (6) | ns | 35 (3) | <0,01 | 12 (7) | ns | 10 (4) | ns | 16 (8) | ns | 12 (6) | ns | 2 (2) | ns | 23 (8) | ns |
| II | 18 (3) | ns | 9 (7) | ns | 47 (5) | <0,05 | 16 (6) | ns | 21 (5) | ns | 25 (8) | ns | 19 (4) | ns | 5 (6) | ns | 16 (10) | ns |
| III | 18 (5) | ns | 12 (5) | ns | 47 (4) | <0,01 | 9 (4) | ns | 24 (3) | <0,05 | 27 (7) | ns | 12 (4) | ns | -1 (4) | ns | 6 (7) | ns |
| aVR | 21 (10) | ns | 2 (5) | ns | 45 (10) | ns | 12 (4) | ns | 18 (8) | ns | 22 (12) | ns | 14 (9) | ns | 1 (1) | ns | 7 (10) | ns |
| aVL | 6 (12) | ns | 9 (6) | ns | 43 (8) | ns | 7 (5) | ns | 13 (5) | ns | 3 (10) | ns | 0 (6) | ns | 3 (2) | ns | 2 (8) | ns |
| aVF | 15 (7) | ns | 8 (6) | ns | 48 (6) | <0,05 | 12 (6) | ns | 23 (2) | <0,001 | 23 (6) | ns | 9 (8) | ns | -1 (1) | ns | 5 (11) | ns |
| V1 | 19 (5) | ns | 14 (5) | ns | 40 (6) | <0,05 | 26 (7) | ns | 20 (2) | <0,05 | 31 (5) | ns | 19 (6) | ns | 1 (1) | ns | 20 (9) | ns |
| V2 | 19 (7) | ns | 13 (5) | ns | 40 (4) | <0,05 | 25 (8) | ns | 13 (3) | ns | 25 (6) | ns | 25 (2) | ns | -3 (9) | ns | 25 (13) | ns |
| V3 | 13 (5) | ns | 12 (4) | ns | 32 (4) | <0,05 | 27 (5) | ns | 2 (6) | ns | 22 (5) | ns | 28 (10) | ns | -2 (1) | ns | 31 (14) | ns |
| V4 | 17 (3) | ns | 12 (3) | ns | 17 (4) | ns | 20 (8) | ns | 13 (4) | ns | 22 (6) | ns | 30 (11) | ns | 1 (1) | ns | 32 (12) | ns |
| V5 | 22 (7) | ns | 14 (3) | ns | 33 (4) | <0,05 | 17 (9) | ns | 19 (3) | ns | 20 (8) | ns | 31 (12) | ns | 2 (1) | ns | 29 (12) | ns |
| V6 | 32 (2) | <0,01 | 13 (7) | ns | 48 (1) | <0,01 | 22 (7) | ns | 25 (5) | ns | 27 (8) | ns | 27 (9) | ns | 0 (1) | ns | 27 (11) | ns |
| V7 | 33 (3) | <0,01 | 13 (7) | ns | 53 (1) | <0,01 | 14 (7) | ns | 24 (4) | ns | 26 (8) | ns | 18 (10) | ns | 1 (4) | ns | 22 (14) | ns |
| V8 | 29 (1) | <0,01 | 7 (4) | ns | 47 (3) | <0,01 | 8 (5) | ns | 23 (5) | ns | 21 (9) | ns | 15 (10) | ns | 2 (2) | ns | 24 (11) | ns |
| V9 | 31 (1) | <0,01 | 7 (4) | ns | 48 (4) | <0,01 | 12 (3) | ns | 24 (4) | ns | 26 (9) | ns | 15 (12) | ns | 0 (1) | ns | 18 (9) | ns |

Values are expressed as mean difference from baseline (standard error of the mean). Abbreviations: LCX: Left circumflex coronary artery; RCA: Right coronary artery LAD: Left anterior descending coronary artery; p: p value of the magnitude of the QRS duration mean difference between the preceeding column and baseline.
