# Supplementary material for: Summation and Cancellation Effects on QRS and ST-Segment Changes Induced by Simultaneous Regional Myocardial Ischemia
Source: Front Physiol. 2018 Apr 3;9:275. doi: 10.3389/fphys.2018.00275 (PMC5891593; doi:10.3389/fphys.2018.00275)
Supplement: Supplementary file 2 [file Table2.DOCX]

**Supplementary table 2. ST-segment deviation values (mV) in each ECG lead in the 12 pigs submitted to single and double coronary artery occlusion**

|  | **GROUP LCX & RCA (n=4)** | | | |  | **GROUP LAD & LCX (n=4)** | | | |  | **GROUP LAD & RCA (n=4)** | | | | |
| --- | --- | --- | --- | --- | --- | --- | --- | --- | --- | --- | --- | --- | --- | --- | --- |
| Leads | LCX | RCA | LCX+RCA | p1 | p2 | LAD | LCX | LAD+LCX | p1 | p 2 | LAD | RCA | LAD+RCA | p 1 | p2 |
| I | 0,06 (0,02) | -0,06 (0,02) | 0,01 (0,01) | 0.02 | ns | -0,05 (0,03) | 0,02 (0,01) | -0,03 (0,01) | ns | ns | 0,01 (0,01) | -0,05 (0,01) | -0,08 (0,01) | 0.00 | ns |
| II | 0,21 (0,06) | -0,05 (0,02) | 0,12 (0,07) | ns | ns | -0,04 (0,03) | 0,2 (0,05) | 0,13 (0,03) | ns | ns | 0,02 (0,01) | 0,05 (0,03) | 0,02 (0.00) | ns | ns |
| III | 0,15 (0,07) | 0,02 (0,02) | 0,13 (0,06) | ns | ns | 0,01 (0,01) | 0,19 (0,04) | 0,19 (0,04) | ns | ns | 0,04 (0,01) | 0,10 (0,03) | 0,08 (0,01) | ns | ns |
| aVR | -0,13 (0,02) | 0,05 (0,02) | -0,06 (0,03) | ns | ns | 0,05 (0,03) | -0,11 (0,03) | -0,04 (0,01) | ns | ns | 0.00 (0,01) | 0,01 (0,02) | 0,02 (0.00) | ns | ns |
| aVL | -0,04 (0,04) | -0,04 (0,01) | -0,06 (0,04) | ns | ns | -0,03 (0,02) | -0,09 (0,02) | -0,11 (0,03) | ns | ns | -0,02 (0.00) | -0,07 (0,02) | -0,06 (0,02) | ns | ns |
| aVF | 0,18 (0,06) | -0,01 (0,02) | 0,13 (0,06) | ns | ns | -0,01 (0,01) | 0,20 (0,05) | 0,16 (0,04) | ns | ns | 0,03 (0,02) | 0,08 (0,04) | 0,06 (0,01) | ns | ns |
| V1 | -0,17 (0,02) | -0,13 (0,01) | -0,43 (0,04) | 0.04 | 0.02 | 0,82 (0,09) | -0,12 (0,03) | 0,53 (0,04) | ns | 0.00 | 0,81 (0,08) | -0,04 (0,11) | 0,62 (0,09) | ns | 0.01 |
| V2 | -0,21 (0,03) | -0,19 (0,03) | -0,53 (0,07) | ns | 0.02 | 0,71 (0,15) | -0,16 (0,03) | 0,38 (0,11) | ns | ns | 0,88 (0,06) | -0,15 (0,04) | 0,6 (0,12) | 0.05 | 0.01 |
| V3 | -0,14 (0,02) | -0,22 (0,04) | -0,46 (0,04) | 0.03 | 0.00 | 0,40 (0,15) | -0,09 (0,04) | 0,16 (0,08) | ns | ns | 0,65 (0,05) | -0,17 (0,02) | 0,39 (0,10) | 0.05 | 0.02 |
| V4 | 0,01 (0,03) | -0,20 (0,03) | -0,24 (0,04) | 0.02 | ns | 0,19 (0,09) | -0,01 (0,04) | 0,08 (0,02) | ns | ns | 0,37 (0,07) | -0,15 (0,01) | 0,15 (0,07) | 0.02 | ns |
| V5 | 0,10 (0,05) | -0,17 (0,03) | -0,08 (0,04) | 0.01 | ns | 0,07 (0,06) | 0,06 (0,03) | 0,07 (0.00) | ns | ns | 0,21 (0,06) | -0,13 (0,02) | 0,03 (0,06) | 0.01 | ns |
| V6 | 0,17 (0,05) | -0,15 (0,02) | -0,01 (0,04) | 0.00 | ns | -0,01 (0,04) | 0,12 (0,04) | 0,07 (0,02) | ns | ns | 0,12 (0,04) | -0,11 (0,02) | -0,03 (0,05) | 0.02 | ns |
| V7 | 0,20 (0,03) | -0,12 (0,02) | 0,06 (0,04) | 0.00 | ns | -0,06 (0,02) | 0,14 (0,03) | 0,07 (0,03) | ns | 0.01 | 0,04 (0,03) | -0,07 (0,01) | -0,06 (0,03) | 0.03 | ns |
| V8 | 0,19 (0,03) | -0,09 (0,01) | 0,07 (0,04) | 0.02 | ns | -0,08 (0,02) | 0,14 (0,03) | 0,05 (0,03) | ns | 0.01 | 0.00 (0,02) | -0,05 (0,01) | -0,06 (0,02) | ns | ns |
| V9 | 0,17 (0,03) | -0,05 (0,01) | 0,09 (0,04) | 0.04 | ns | -0,08 (0,02) | 0,12 (0,03) | 0,04 (0,03) | ns | 0.04 | -0,02 (0,01) | -0,03 (0,01) | -0,06 (0,01) | ns | ns |

Values are expressed as mean and (standard error of the mean). Abbreviations: LCX: Left circumflex coronary artery; RCA: Right coronary artery LAD: Left anterior descending coronary artery; p1: p value for the comparison between the ST segment values of the first column (single occlusion) and the third column (double occlusion) in each experimental group; p2: p value for the comparison between the ST segment values of the second column (single occlusion) and the third column (double occlusion) in each experimental group
